# Health care for under-fives in Ile-Ife, South-West Nigeria: Effect of the Integrated Management of Childhood Illnesses (IMCI) strategy on growth and development of under-fives

**DOI:** 10.4102/phcfm.v1i1.29

**Published:** 2009-06-30

**Authors:** Olufunke M. Ebuehi

**Affiliations:** 1 Department of Community Health and Primary Care, University of Lagos, Nigeria

**Keywords:** caregivers, Community-Integrated Management of Childhood Illnesses, growth promotion and development practices, paediatrics, Ile-Ife

## Abstract

**Background:**

The study obtained information on key growth promotion and developmental household and community health practices in Community-Integrated Management of Childhood Illnesses (C-IMCI) and non-C-IMCI in local government areas (LGAs) in Osun State, Nigeria, to determine the differences that existed, between these LGAs.

**Method:**

A cross-sectional comparative study to compare Integrated Management of Childhood Illnesses (IMCI) key growth promotion and development health practices in two LGAs in Osun State was conducted using quantitative and qualitative techniques. Data analysis was done using Epi Info version 6.0 for the quantitative survey and a content analysis method for the qualitative survey. The subjects were mothers or caregivers of children 0–59 months of age, and their index children.

**Results:**

Findings revealed that the IMCI key growth and development health practices were generally better rated in the CIMCI-compliant LGA than in the non-CIMCI compliant LGA. Breastfeeding practice was widespread in both LGAs. However, the exclusive breastfeeding (EBF) rate among children under six months was higher in the compliant LGA (66.7%) than in the non-compliant LGA (25%). More caregivers (59.7%) from the non-compliant LGA introduced complementary feeds earlier than six months. Growth monitoring activities revealed that there were more underweight children (19.1%) in the non-compliant LGA. Community Resource Persons (CORPs) and health workers were the most popular sources of information on IMCI key practices in the compliant LGA, while in the non-compliant LGA the traditional healers, elders and, to a lesser extent, health workers provided information on these key practices.

**Conclusion:**

The IMCI strategy, if well implemented, is an effective and low-cost intervention that is useful in achieving optimal growth, development and survival of Nigerian children.

## INTRODUCTION

The level of under-five mortality in the less developed countries of the world, especially in sub-Saharan Africa, remains very high despite enormous investments in health system reforms and several vertical programmes.^1^ Each year almost 11 million children in low- and middle-income countries die before they reach their fifth birthday.^2^ Five in 10 of these deaths are due to just five conditions: malaria, pneumonia, diarrhoea, measles and HIV – often in combination.^3^ Malnutrition contributes to over 60% of these deaths.^4^ The Integrated Management of Childhood Illness (IMCI) is a strategy developed by the World Health Organization (WHO), the United Nations Children's Fund (UNICEF) and other technical partners to address major child health problems in the developing world.^5^ IMCI seeks to address these problems through three intervention strategies – improved case-management, improved health systems support, and improved family and community practices.

Success in reducing childhood mortality and in promoting optimal growth and development of children requires more than the availability of health services with well-trained health personnel. Since a major concern and responsibility of parents is caring for their children, success requires a partnership between health workers and families, and the support from their communities. Health workers in partnership with other developmental agencies need to work together with the families and solicit community support to ensure improved health practices for childcare at home, home treatment of mild illnesses with traditional non-harmful home remedies, timely recognition and prompt health care seeking when the child is sick, and compliance to the treatment.^6^

In 1996, Nigeria expressed willingness to implement the IMCI strategy and in 1997 gave a firm commitment to its adoption and implementation. In order to gain initial experience in IMCI implementation the government commenced with implementation of the first two components in six local government areas (LGAs) spread across the country's six geopolitical zones. Later, in 2005, the community component was implemented in the same LGAs. Community Resource Persons (CORPs) were trained to provide information about the key practices to caregivers and to ensure that they adopted the practices.

This study obtained information on key growth and development household and community practices in C-IMCI and non-C-IMCI LGAs in Osun State, Nigeria, in order to determine what differences existed in these LGAs. Information derived will be useful to assess the achievement of the objectives of the community IMCI strategy over time. Information obtained may also be useful in advocating for expansion to other areas within Nigeria that are not yet C-IMCI compliant.

## METHOD

**Study sites:** The study was conducted in one C-IMCI and one non-C-IMCI implemented LGAs in Osun State, Nigeria, between August and September 2007. Osun State is an inland state located in the south-western zone of Nigeria. Its capital is Osogbo. Osun State, with a population of about 3.4 million,^7^ has 30 local government areas.

The study site of Ife Central was purposively chosen because it is the only LGA in the south-western zone of Nigeria that is already C-IMCI compliant. Ife North was selected amongst the remaining 29 non C-IMCI compliant LGAs by the balloting system. The two LGAs are a mixed blend of rural and urban communities, with the majority of the population involved in farming and trading in local produce.

**Study design:** A cross-sectional design was used in the survey.

**Study population:** The study subjects were mothers/caregivers of children 0–59 months of age, and their index children.

**Sampling technique:** A multi-stage sampling procedure was used to select 260 and 255 respondents from the compliant (Ife Central) and non-compliant (Ife North) LGAs respectively, as described below:

***Stage 1***: Enumerated areas (EAs) of the two intervention wards (wards 4 and 5) and those of two randomly selected wards (wards 6 and 9) in the control site were used as the primary sampling units. 2% of the EAs in each LGA were selected using the systematic random sampling method. At each site the sample sizes of 260 and 255 were divided by the selected number of EAs to determine the number of respondents that were to be recruited from each EA. Ife Central has 180 EAs; four EAs were randomly selected and 65 respondents from each EA were recruited. Ife North has 163 EAs; three EAs were randomly selected and 85 respondents from each EA were recruited.

***Stage 2:*** In the two LGAs, all the streets in the selected EAs were systematically labelled (high) ‘H’ or (low) ‘L’ (as secondary sampling units), and one street was randomly selected (using the ballot system) from each EA. Interviews began with people from eligible households in the low-numbered houses and continued towards those in the high-numbered houses for an ‘L’ street, and in the reverse order for an ‘H’ street.

***Stage 3:*** The sampling unit was the household. In any visited household the eligibility for participation was the presence of a child under 5 years of age (i.e. 0–59 completed months). In cases where the household had more than one such subject a ballot system was adopted to select the reference child.

### Data collection techniques and instruments

#### Structured questionnaire

An interviewer administered a 23-page structured questionnaire (originally developed by WHO/Nigeria^8^, and adapted and used for eliciting information from caregivers of eligible children). The instrument covers the four key areas of household practices, viz: growth promotion, disease prevention, home management and care seeking. The instruments were pre-tested in Lagos State and amended as appropriate.

#### Weighing of reference children

Standardised and calibrated stand-on scales were used to weigh the children in their underpants. In the case of children who could not stand, the mother was weighed together with the child, then the mother was weighed alone and the difference recorded as the child's weight.

Evidence of immunisation was obtained by sighting the immunisation card or by history. Vitamin A coverage rate was sought by asking caregivers whether or not their children had received Vitamin A capsules and also by checking the immunisation/child health card. A sample of the Vitamin A capsule commonly used was shown to the respondents to aid recall and eliminate confusion with oral polio vaccine. Information was also obtained on what caregivers did during their child's last illness.

**Quantitative data management** Completed questionnaires were checked for completeness, errors and inconsistencies; those detected were verified by the interviewers concerned.

The Epi Info software (version 6.04c) was used for data entry, validation, cleaning and analysis. The prevalence of key practices were calculated. Frequency distributions were generated for all categorical variables and means and standard deviations were determined for continuous variables. Anthropometric measurements were converted, using EpiNut, to standard z-scores. ‘Underweight’ was defined as z-scores of –2 or less for the WHO Child Growth Standards reference value for age (National Center for Health Statistics^9^). The chi-square test was used to compare categorical variables for possible significant differences between compliant and non-compliant LGAs^10^. All statistical tests were carried out at a 0.05% level of significance, at a 95% level of confidence.

### The qualitative study

Qualitative methods were used to provide additional explanations to some of the findings that emerged from the quantitative survey, and to identify the use or non-use of key household practices by caregivers as well as to provide information on community structures that support child health and inter-sectoral linkages.

**Study participants:** These consisted of community representatives and community based opinion leaders from the two LGAs and LGA officials.

**Data collection methods:** These comprised Focus Group Discussions (FGDs) and in-depth interviews of key informants in the community using the FGD guide and the in-depth interview guide^11^.

**Data collection instruments:** FGD and in-depth interview guides adapted from those designed by the Federal Ministry of Health, IMCI unit, in collaboration with the WHO, were used. The instruments were pre-tested in Lagos State and amended as appropriate.

### Ethical consideration

Approval was obtained from the Research and Ethics Committee of the Lagos University Teaching Hospital. Written informed consent was obtained from study participants.

## RESULTS

242 and 246 valid questionnaires were collected from the LGAs, yielding response rates of 93.1% and 96.5% respectively. The mean ages of caregivers from CIMCI and non-CIMCI compliant LGAs were 29.8 + 6.9 and 31.5 + 9.4 respectively (p = 0.046). The majority were married females. Most were mothers and constituted the majority of day and night time caregivers in both LGAs. Less than one tenth in both LGAs had no formal education. More than half of them had completed secondary education and higher (Table 1). The ages of the index children from the CIMCI compliant LGA ranged from 2 to 57 months, while those from the non-CIMCI LGA ranged from 1 to 59 months. The mean ages of index children from compliant and non-compliant LGAs were 23.1 + 12.3 and 18.4 + 13.6 respectively. Slightly more than half of the total numbers of children from the two LGAs were males (Table 1).

**TABLE 1:** Demographic characteristics of respondents and index children

	C-IMCI COMPLIANT LGA n (%)	NON-C-IMCI COMPLIANT LGA n (%)	X^2^	P
**DEMOGRAPHICS OF CAREGIVERS**	**N = 242**	**N = 246**		
**Age (years)**				
10–19	1 (0.4)	3 (1.2)		
20–29	131 (54.2)	132 (53.7)		
30–39	86 (35.5)	74 (30.1)	11.29	0.046
40–49	18 (7.4)	15 (6.1)		
50–59	4 (1.7	15 (6.1)		
60–69	2 (0.8)	7 (2.8)		
**Sex**				
Male	2 (0.8)	10 (4.1)	5.33	
Female	240 (99.2)	236 (95.9)	0.021	
**Marital status**				
Single	54 (22.3)	48 (19.5)		
Married	173 (71.5)	179 (72.8)	12.39	
Divorced	6 (2.5)	1 (0.4)	0.015	
Separated	8 (3.3)	7 (2.8)		
Widow/Widower	1 (0.4)	11 (4.5)		
**Educational status**				
None	18 (7.4)	22 (8.9)		
Quranic school	11 (4.5)	18 (7.3)		
Primary not completed	21 (8.7)	27 (11)	10.31	0.112
Primary completed	42 (17.4)	48 (19.5)		
Junior secondary	55 (22.7)	56 (22.8)		
Senior secondary	76 (31.4)	69 (28.1)		
Post secondary	19 (7.9)	6 (2.4)		
**DEMOGRAPHICS OF CAREGIVERS’ INDEX CHILDREN**	**N = 242**	**N = 246**		
**Age (months)**				
0-5	27 (11.2)	8 (3.3)		
6-11	69 (28.5)	43 (17.5)		
12-23	83 (34.3)	92 (37.4)	29.46	0.000
24-35	24 (9.9)	54 (22.0)		
36-59	39 (16.1)	49 (19.8)		
**Sex**				
Male	130 (53.7)	125 (50.8)		
Female	112 (46.3)	121(49.2)	0.41	0.520

### Growth promotion and development practices

#### Breastfeeding

More children under 6 months (66.7%) from the compliant LGA were exclusively breastfed than the same age group (25%) from the non-compliant LGA (p = 0.037). A similar trend was observed in children aged 12–23 and 20–23 months (Table 2). More children (93.9%) under two years of age from the compliant LGA were reported to have been given colostrum than their counterparts (44.1%) from the non-compliant LGA. Of the respondents who did not give colostrum, the reasons were that ‘it was not good for the baby’ (77.8% and 68.8%) and that ‘it made the child sick’ (22.2% and 31.2%) in the compliant and non-compliant LGAs respectively (p = 0.37) (Table 3). The pre-lacteal feeding rate was higher in the non-compliant (53.9%) than in the compliant LGA (15.7%), p < 0.01). Water alone was the most common pre-lacteal drink given in both LGAs (Table 3). Virtually all the children aged 6–9 months in the two LGAs had commenced complementary feeding. However, more caregivers (59.7%) from the non-compliant LGA introduced complementary feeds earlier than 6 months compared to those (28.9%) from the compliant LGA (p = 0.000) (Table 4).

**TABLE 2:** Children under six months that were exclusively breastfed and children aged 12-23 months and 20-23 months still breastfeeding

	PRE-CIMCI n (%)	C-IMCI COMPLIANT LGA n (%)	NON-CIMCI COMPLIANT LGA n (%)	X^2^	P
**EBF**	**N = 97**	**N = 27**	**N = 8**		
Yes	19 (19.6)	18 (66.7)	2 (25)	4.38	0.037
No	78 (80.4)	9 (33.3)	6 (75)		
**BREASTFEEDING CHILDREN**					
**Aged 12–23 (months)**	**N = 142**	**N = 82**	**N = 83**		
Yes	71 (50.0)	71 (86.6)	32 (38.6)	40.6	0.000
No	71 (50.0)	11 (23.4)	51 (61.4)		
**Aged 20–23 (months)**	**N = 31**	**N = 20**	**N = 24**		
Yes	5 (16.1)	13 (65)	0 (0)	22.1	0.000
No	26 (83.9)	7 (35)	24 (100)		

Note: X^2^ and P values represent comparisons of data between compliant and non-compliant LGAs after CIMCI intervention

**TABLE 3:** Caregivers who reported giving colostrum and pre-lacteal feeds, and the type of pre-lacteal feeds/drinks given

	PRE-CIMCI n (%)	C-IMCI COMPLIANT LGA n (%)	NON-CIMCI COMPLIANT LGA n (%)	X^2^	P
**GAVE COLOSTRUM**	**N = 465**	**N = 179**	**N = 143**		
Yes	397 (85.4)	168 (93.9)	63 (44.1)	97.2	0.000
No	68 (14.6)	11 (6.1)	80 (55.9)		
**PRE-LACTEAL FEED**	**N = 91**	**N = 179**	**N = 143**		
Yes	18 (19.8)	28 (15.7)	77 (53.9)	52.8	0.000
No	72 (79.1)	151(84.3)	66 (46.1)		
**TYPES OF PRE-LACTEAL FEEDS/DRINKS GIVEN**	**N = 29**	**N = 28**	**N = 77**		
Water alone	23 (79.3)	23 (82.1)	56 (72.7)		
Glucose water	3 (10.3)	4 (14.3)	20 (26)	0.98	0.32
Herbs	3 (10.3)	1 (3.6)	1 (1.3)		

**TABLE 4:** Age at introduction of, and methods of, complementary feeding in children under 24 months

	CIMCI COMPLIANT LGA n (%)	NON-CIMCI COMPLIANT LGA n (%)	X^2^	P
**AGE (IN MONTHS) AT INTRODUCTION OF COMPLEMENTARY FEEDING**	**N = 173**	**N = 134**		
< 3	33 (19.1)	60 (44.8	38.86	0.0000
3–5	17 (9.8)	20 (14.9)		
6–8	114 (65.9)	41 (30.6)		
Above 8	9 (5.2)	13 (9.7)		
**METHODS OF FEEDING**	**N = 171**	**N = 134**		
Bottle	13 (7.6)	23 (17.2)	10.94	0.0042
Cup/plate and spoon	154 (90.1)	102 (76.1)		
Forced feeding	4 (2.3)	9 (6.7)		

#### Types of complementary feeds

For children aged 6–9 months, porridge (pap) was the most popular complementary feed used. Other types of complementary feeds mentioned included: beans in different forms (akara, moi-moi), yam flour (amala), yam, rice, meat and fish. The main additives to the pap were sugar, milk, soy milk, groundnut and crayfish in both LGAs. In neither of the LGAs did the caregiver give pap alone. The most common method of feeding in both LGAs was feeding with cup/plate and spoon; however, bottle feeding and forced feeding rates were higher in the non-CIMCI compliant LGA (Table 4).

#### Growth monitoring

Fewer index children (42.8%) from the non-compliant than those (65.8%) from the compliant LGA had immunisation/child health cards. Evidence of growth monitoring activity through weight charting revealed that of the 65.8% and 42.8% children whose cards were seen, in the compliant and non-compliant LGAs respectively, 24.4% and 24.1% had no weight records. The Vitamin A supplementation rate in children aged 6–59 months was higher in the compliant than in the non-compliant LGA (Table 5).

**TABLE 5:** Index children with immunisation/child health cards and under-fives with weight charting and Vitamin A supplementation (card + recall) in the 6 months preceding the study

	C-IMCI COMPLIANT LGA n (%)	NON-CIMCI COMPLIANT LGA n (%)	X^2^	P
**IMMUNISATION/ CHILD HEALTH CARDS**	**N = 237**	**N = 243**		
Yes	156 (65.8)	104 (42.8)	25.6	0.000
No	81 (34.2)	137 (56.4)		
Don't know	0 (0.0)	2 (0.8)		
**NUMBER. WEIGHT RECORDS**	**N = 156**	**N = 108**		
0	38 (24.4)	26 (24.1)	1.37	0.85
1	23 (14.7)	19 (17.6)		
2	38 (24.4)	30 (27.8)		
3	38 (24.4)	21(19.4)		
4	19 (12.2)	12 (11.2)		
**EVER HAD VITAMIN A**	**N = 220**	**N = 241**		
Yes	138 (62.7)	74 (30.7)	47.5	0.000
No	75 (34.1)	165 (68.5)		
Don't know	7 (3.2)	2 (0.8)		

#### Prevalence of childhood disorders

More children from the non-compliant LGA had diarrhoea and cough two weeks preceding the study (p = 0.014 and p = 0.000 respectively). However, during the same period more children from the compliant LGA had fever (p = 0.48).

#### Prevalence of ‘low weight for age’ among index children by LGA

More children from the non-compliant LGA had low weights for their ages. For all the age groups there were more ‘low weight for age’ children from the non-compliant than the compliant LGA. More females than males were underweight in the compliant LGA, while more males than females were underweight in the non-compliant LGA (Table 6).

**TABLE 6:** Prevalence, age and sex distribution of ‘low weight for age’ among index children by LGA

	Pre-CIMCI n (%)	C-IMCI COMPLIANT LGA n (%)			NON-C-IMCI COMPLIANT LGA n (%)			X^2^	P
**INDEX CHILD HAD ‘LOW WEIGHT FOR AGE’**	**N =511**	**N = 242**			**N = 246**				
Yes	102 (20.0)	29 (12)			47 (19.1)			4.71	0.030
No	409 (80.0)	213 (88)			199 (80.9)				
**AGE (MONTHS)**		**LOW WEIGHT FOR AGE**			**LOW WEIGHT FOR AGE**				
					
		**YES**	**NO**	**TOTAL**	**YES**	**NO**	**TOTAL**		
					
		**n (%)**	**n (%)**	**n (%)**	**n (%)**	**n (%)**	**n (%)**		
< 12		18 (18.8)	78 (81.2)	96 (100)	17 (33.3)	34 (66.7)	51 (100)	3.90	0.048
12-23		11 (13.3)	72 (86.7)	83 (100)	15 (16.3)	77 (83.7)	92 (100)	0.32	0.571
24-35		0 (0.0)	24 (100)	24 (100)	10 (18.5)	44 (81.5)	54 (100)	5.10	0.024
36-47		0 (0.0)	31 (100)	31 (100)	3 (8.6)	32 (91.4)	35 (100)	2.78	0.095
48-59		0 (0.0)	8 (100)	8 (100)	2 (14.3)	12 (85.7)	14 (100)	1.26	0.262
**STATUS**		**MALE**	**FEMALE**	**TOTAL**	**MALE**	**FEMALE**	**TOTAL**		
					
		**n (%)**	**n (%)**	**n (%)**	**n (%)**	**n (%)**	**n (%)**		
**UNDER-WEIGHT**		13 (10)	16 (14.3)	29 (12)	36 (28.8)	11 (9.1)	47 (19.1)		
**NORMAL**		117 (90)	96 (85.7)	213 (88)	89 (71.2	110 90.9)	199 (80.9)		

**TOTAL**		**130 (100)**	**112 (100)**	**242 (100)**	**125 (100)**	**121 (100)**	**246 (100)**		

* X^2^ = 1.05 P = 0.31 * X^2^ = 15·5 p = 0.000

*statistical values for sex distribution of ‘low weight for age’ children

#### Sources of information on home management of childhood illnesses

More caregivers (78.5%) from the compliant LGA than those (30.5%) from the non-compliant LGA obtained information about home management of childhood illnesses from community based workers (p = 0.000). About two thirds of caregivers from the non-compliant LGA obtained this information from relatives.

## FINDINGS OF THE QUALITATIVE STUDY

### Key preventive and health promotional practices of childcare

#### Exclusive breastfeeding

Awareness about exclusive breastfeeding (EBF) was high in both LGAs as the majority of the caregivers had heard about EBF and could explain its importance. One female participant in the compliant LGA said: *‘*It is giving breast milk without water or other things for six months.’ Another female participant from the same LGA said:

*It makes the child to be* [sic] *strong and protects the child from watery stool, cough and catarrh. It also helps the mother's body to recover quickly after delivery and helps to prevent pregnancy*.

A female participant from the non-compliant LGA expressed concerns about the difficulty of introducing complementary feeds. She said:

*I agree that breast milk alone is good for baby, but I notice that most babies on breast alone refuse to take other things when it is time to give them other food. They just want to continue the breast milk, which is not sufficient for them*.

#### Oral rehydration therapy

Most caregivers in the compliant LGA and a few in the non-compliant LGA knew the meaning and use of oral rehydration therapy (ORT). Most participants in the compliant and very few in the non-compliant LGA could accurately describe the preparation of salt sugar solutionand used ORTfor treating childhood diarrhoea. A female participant in the compliant LGA said: ‘We give our children salt/sugar solution when they have diarrhoea. We either buy it from the chemist or make it at home. We give it to the children and the watery stool stops’. A female participant in the non-compliant LGA said: ‘I've heard about the “water” but I can't really remember how to make it. The nurse at the health centre told us about it. When my child has watery stools, I give him his food and water and he gets well.’

#### Immunisation

In both LGAs, awareness and benefits of immunisation were high. All respondents reported taking their children for immunisation and the CORPs ensured that the children completed their immunisation by regularly reminding mothers to take their children for immunisation. In the non-compliant LGA, however, although mothers did strive to take their babies for immunisation, they sometimes defaulted due to the long distances from their homes to the nearest PHC centres, as evident from the higher dropout rate in this LGA. One female participant said:

*We like to take our children for immunisation, but the clinic is too far! We know the immunisation is to help them. Please, we would like it if they can come to the town hall on some days to give the immunisation*.

The Primary Health Care Director for the non-compliant LGA reported that caregivers were mistaking the National Immunisation Days (NIDs) for the routine immunisation, and hence stopped bringing their children to the health centres for immunisation. NIDs are specific days to ‘mop up’ children that have not had the opportunity of being immunised in health facilities. Specific days are set up quarterly to take care of these children, and immunisation is done from house to house. Routine immunisation, on the other hand, is a health facility based immunisation program. Mothers are expected to bring their babies for all the vaccines listed on the national schedule. The apparent lack of understanding of the difference between these immunisation programs necessitated the need to reach caregivers through the community ‘social mobilisation committee’ to explain the difference. Subsequently, the turnout rate for routine immunisation gradually increased.

#### Harmful traditional practices and child survival

In the compliant LGA, the practice of not giving colostrum to babies was reported to have decreased considerably with IMCI implementation. Also, giving newborns a herbal mixture as a way of ‘intestinal cleansing’ has also stopped. One female participant said:

*Before, we used to give our babies water and glucose before we start them on breast milk but this has stopped, since we were told at the health centre that we should give only breast milk. We also gave herbs to clean the baby's stomach before breastfeeding, because the ‘black stool’ the baby passes when it is born shows that the baby's stomach is dirty. This practice is reducing now, since we were told that the herbs can harm our babies*.

However, in the non-compliant LGA some caregivers still did not give colostrum to their newborns, due to the belief that it is harmful to the baby. Some families still believe in applying scarification marks to their children for clan identification.

Participants from both LGAs reported that female genital mutilation was no longer practised. A male participant in the compliant LGA said ‘*Aha aha (laugh)!! We don't cut our female children anymore! We now know better that it doesn't give any benefit to the female child!’*

#### Health facilities that support child health care

In both LGAs the structures used to support child health care are government and mission hospitals (Seventh Day Adventist and St Andrew's Clinic), chemists, and the use of herbs. In the non-compliant LGA, often clients who visited government health facilities had to travel long distances and then had to wait for long hours to receive services. The cost (direct and indirect) of care also discouraged utilisation of these services. A heavy dependence on traditional methods of childcare was still very evident. However, in the compliant LGA, government health centres provided relatively effective, efficient and affordable (free) health care services for children, and hence recorded high patronage.

#### Community structures that support child health care

Community structures offering child health care services exist in both LGAs. In the compliant LGA, 60 CORPs trained in community IMCI provide information to caregivers on child survival household and community practices. Regular home visits are made within these communities to ensure adoption of these practices and also to identify children needing care outside the home, and to refer promptly. Meetings are held twice a month to deliberate on issues affecting child health. Each CORP receives a monthly stipend of one thousand naira (N1000). In the non-compliant LGA, the ‘social mobilsation committees’ exist. Their responsibilities include mobilising communities for NIDSs, routine immunisation and other issues related to community welfare, such as the provision of water and improved sanitation.

An opinion leader in this LGA said:

*In this place, health workers come around to attend to our children but they can't cover all the areas. We appeal to government to give us more health workers. We are ready to work with government to make sure that our people enjoy good health*.

Other community structures that supported childcare practices were the radio, television, chemists, elders and NID programmes.

#### Community based health information system

The compliant LGA had an established community based health information system of birth registration. CORPs, family heads and elders collect information on births within the communities. This information is then forwarded to the National Population Commission. In the non-compliant LGA, such a system does not exist but it expressed a desire to set up one with the support of the local government.

## DISCUSSION

The present study has demonstrated the benefits of early interventions in C-IMCI in the compliant LGA. When comparing baseline findings^8^ in the compliant LGA with those from this study, some of the outcome indicators were better rated after CIMCI implementation. For example, the prevalence of some IMCI targeted disorders was reduced after implementation. The prevalence of diarrhoea and cough were reduced from baseline values of 13% and 29.9% to 9.5% and 18.6%, respectively. The proportion of underweight children was also reduced from a baseline value of 20% to 12% in the compliant LGA. Similar trends were also observed for some key practices, such as exclusive breastfeeding (breastfeeding for the recommended duration of 24 months).

Comparing the present findings in both LGAs, the compliant LGA had relatively better results than the non-compliant LGA; caregivers in this LGA demonstrated better knowledge and improved practices than those of the comparative LGA. In spite of these achievements, there are still gaps to be filled in the compliant LGA. The prevalence of fever did not change significantly (p = 0.37) from a baseline value of 36.2% to 30.6% after intervention. This may, however, not be unrelated to the scarcity of ‘high cost’ insecticide treated nets (ITNs) and the non-use of other preventive methods of malaria.

Potential openings for interventions in the non-compliant LGA exist. This is evident in opinion leaders’ calls for the establishment of the IMCI programme in the LGA and their willingness to collaborate with government on measures to improve the health of the community. This must be harnessed if the millennium goals 4 and 5 are to be achieved by 2015.^12^

A significantly higher (p < 0.01) EBF rate in the compliant LGA than in the non-CIMCI compliant LGA is in agreement with the findings of Haider *et al*.^13^ and Nankunda who observed that using community based peer counsellors increased EBF rates in Bangladesh and rural Uganda. As indicated in this study, a high awareness of EBF exists, but the challenge is with the perceived necessity to give water to the baby, based on the misconception that a baby cannot eat without taking water. It is said that infants aged 0–5 months who are not exclusively breastfed are known to have a seven- and five-fold increased risk of dying from diarrhoea and pneumonia respectively, in comparison to those exclusively breastfed.^14^ Developing Information, Education and Communication (IEC) material that emphasise the sufficiency of the nutrients and water in breast milk is therefore imperative.

More than half of the caregivers (55.6%) in the non-compliant LGA did not give colostrum to their newborns because of the misconception that it is not good for babies. This is of great concern, as colostrum is known to be very rich in anti-infective agents that protect the newborn from common childhood infections. A high pre-lacteal feeding rate of 53.9% in the non-compliant LGA explains the strong resistance to the idea of not giving water to newborns.

IEC material emphasising the highly protective contents of colostrum and the harmful effects of pre-lacteal feeds should be developed. These packages must be culturally acceptable, and developed for the education of community members with particular focus on men and women of all ages, including the post-reproductive age group.

Complementary feeding practices revealed that more than half (59.7%) of caregivers from the non-compliant LGA introduced complementary feeds earlier than 6 months compared with 28.9% of their counterparts from the compliant LGA. These findings agree with those of Vaahtera et al.^15^, who observed that, in rural Malawi, feeds were introduced at median ages of 2.5 and 6.3 months (much earlier than recommended). Again it is clear that the introduction of complementary feeding earlier than recommended has far-reaching implications for child survival, growth and development, as it denies the child the full benefits of EBF, and predisposes the child to increased risk of infection and malnutrition, amongst others. Pap was the commonest complementary feed, but it was of thin consistency in more than half (57.8%) of the respondents, particularly in the non-compliant LGA. This practice provides poor quality food for a child at a period of rapid growth and brain development, as was evident from a randomised, community based trial of Lartey et al.^16^ in Ghana, where it was observed that infants (between the ages of 6–12 months) fed with ‘fortified koko’ (a fermented maize porridge) had improved growth (better iron and vitamin A status) relative to the non-intervention group. This probably explains why there was a relatively higher proportion of underweight children (19.1%) in the noncompliant LGA.

The practice of giving poor quality food to children could be improved upon through educating communities on the timely introduction of appropriate quantity and quality of complementary foods, as seen in the findings of an effectiveness trial of community nutritional counselling in India^17^ which showed improvements in the knowledge of caregivers in childcare and complementary feeding practices.

In cases where there was a relatively lower proportion of children with health cards and irregular weighing there could be delays in the recognition of underweight children and malnutrition in children. Similar findings were observed by Mukanga and Kiguli^18^ in Uganda, who found that 66% of children aged 0–24 months had child health cards and those with health cards were more likely to be up to date with immunisation and growth monitoring.

A higher prevalence of some IMCI targeted disorders such as diarrhoea and cough was observed in the non-compliant than in the compliant LGA. This may be due to the relatively poor knowledge of caregivers in this LGA about the causes and appropriate home management of these disorders (74.4% and 59.8% attributed malaria and diarrhoea to sunlight/hard work and teething, respectively, and only 18.4% of caregivers used ORS in the treatment of diarrhoea). Studies have shown that using community based interventions significantly reduces morbidity and mortality in the under-fives.^19, 20, 21, 22^, ^23^ A hygiene education intervention in Zaire addressing the disposal of children's faeces and hand washing after defecation, before meal preparation and eating, resulted in an 11% reduction in the risk of diarrhoea during the peak diarrhoeal season.^2^

A higher proportion of children (30.6%) from the compliant than the non-compliant LGA (27.6%) had fever two weeks prior to the study. This may be due to the very low use of ITNs in both LGAs, particularly in the non-complaint LGA. Reasons for this differed between the LGAs. High cost was the major barrier to use in the compliant LGA, while ignorance was responsible for its non-use in the non-compliant LGA.

The fact that the health worker remained the single most common source of information on common childhood disorders implies that a regular appraisal of their counselling skills is required, which should be improved through training. A notable observation was that almost 80% of caregivers from the compliant LGA had also obtained information on growth promotion and home management of childhood illnesses from trained CORPs, which showed the synergistic effect of the community IMCI intervention in building caregivers’ capacity in C-IMCI.

Intersectoral collaborative efforts existing between different departments (such as education, health, agriculture, information and water and sanitation) in both LGAs should be strengthened. This should ensure the achievement and sustainability of the objectives of the IMCI. Non-functional development associations should be renewed.

In conclusion, the findings of this study have revealed the positive effect of partnering with communities in improving their health status. Partnering promotes a sense of belonging and ownership among communities, thereby ensuring the sustainability of developmental programmes. The fact that some gaps still exist in the compliant LGA underscores the need to empower communities with regular and appropriate health care information, and resources to improve on their existing perceptions and practices, particularly with respect to childcare practices.

The success story in the C-IMCI compliant LGA justifies the need for the scaling up of C- IMCI to other communities that are yet to commence implementation. The gains recorded in the compliant LGA should be consolidated by strengthening existing partnerships between government and these communities.

Once the strategy is correctly implemented, it can be safely assumed that the stage will be set for reducing deaths and disabilities among the under-fives, caused by the IMCI-targeted preventable diseases. The IMCI strategy, if well implemented, is an effective and low-cost intervention that is useful in achieving optimal growth, development and survival of Nigerian children.
